# Genome structure of cotton revealed by a genome-wide SSR genetic map constructed from a BC_1 _population between *gossypium hirsutum *and *G. barbadense*

**DOI:** 10.1186/1471-2164-12-15

**Published:** 2011-01-09

**Authors:** Yu Yu, Daojun Yuan, Shaoguang Liang, Ximei Li, Xiaqing Wang, Zhongxu Lin, Xianlong Zhang

**Affiliations:** 1 National Key Laboratory of Crop Genetic Improvement & National Centre of Plant Gene Research (Wuhan), Huazhong Agricultural University, Wuhan 430070, Hubei, PR China; 2 Cotton Institute, Xinjiang Academy of Agriculture and Reclamation Science, Shihezi 832000, Xinjiang, PR China

## Abstract

**Background:**

Cotton, with a large genome, is an important crop throughout the world. A high-density genetic linkage map is the prerequisite for cotton genetics and breeding. A genetic map based on simple polymerase chain reaction markers will be efficient for marker-assisted breeding in cotton, and markers from transcribed sequences have more chance to target genes related to traits. To construct a genome-wide, functional marker-based genetic linkage map in cotton, we isolated and mapped expressed sequence tag-simple sequence repeats (EST-SSRs) from cotton ESTs derived from the A_1_, D_5_, (AD)_1_, and (AD)_2 _genome.

**Results:**

A total of 3177 new EST-SSRs developed in our laboratory and other newly released SSRs were used to enrich our interspecific BC_1 _genetic linkage map. A total of 547 loci and 911 loci were obtained from our EST-SSRs and the newly released SSRs, respectively. The 1458 loci together with our previously published data were used to construct an updated genetic linkage map. The final map included 2316 loci on the 26 cotton chromosomes, 4418.9 cM in total length and 1.91 cM in average distance between adjacent markers. To our knowledge, this map is one of the three most dense linkage maps in cotton. Twenty-one segregation distortion regions (SDRs) were found in this map; three segregation distorted chromosomes, Chr02, Chr16, and Chr18, were identified with 99.9% of distorted markers segregating toward the heterozygous allele. Functional analysis of SSR sequences showed that 1633 loci of this map (70.6%) were transcribed loci and 1332 loci (57.5%) were translated loci.

**Conclusions:**

This map lays groundwork for further genetic analyses of important quantitative traits, marker-assisted selection, and genome organization architecture in cotton as well as for comparative genomics between cotton and other species. The segregation distorted chromosomes can be a guide to identify segregation distortion loci in cotton. The annotation of SSR sequences identified frequent and rare gene ontology items on each chromosome, which is helpful to discover functions of cotton chromosomes.

## Background

Cotton (*Gossypium spp*.) is the most important fiber crop in the world and one of the most important oilseed crops. Within the genus *Gossypium*, two cultivated allotetraploid species, *G. hirsutum *L. and *G. barbadense *L., account for 90% and 8% of the world cotton production respectively [1]. The construction of a molecular genetic map is a foundation in genetic dissection of economically important traits, marker-assisted selection (MAS), and map-based cloning. It provides new insights into genome structure and chromosomal architecture of the cotton genome. However, the allotetraploid (2n = 4x = 46) species has a large genome size of ~ 2246 Mb [2], which has hindered the development of a high-density map.

To explore the cotton genome structure and to identify quantitative trait loci (QTLs) for agronomically important traits which can facilitate MAS in cotton, several genetic maps have been constructed including high-density interspecific [3-8] and intraspecific maps [9-13]. The early cotton genetic maps were comprised of only restriction fragment length polymorphisms (RFLPs) [2]; later, polymerase chain reaction (PCR)-based markers were widely adopted, including random amplified polymorphic DNA (RAPD), amplified fragment length polymorphism (AFLP), simple sequence repeat (SSR), sequence-related amplified polymorphism (SRAP), and target region amplification polymorphism (TRAP) [1]. Among the molecular markers, SSRs have become popular markers of choice for cotton genetic analysis and mapping.

SSRs, consisting of a variable number of tandem repeats, are mainly characterized by their high frequency, even distribution, co-dominance, reproducibility, and high polymorphism [14,15]. Because of these characteristics, microsatellites have become the most favoured genetic markers for plant breeding and genetics such as genetic diversity assessment, genetic map construction, QTL mapping, and marker aided selection, etc [16]. In general, SSRs are identified from either genomic DNA or cDNA sequences. The usual source sequences for SSRs have included SSR-enriched library clones, expressed sequence tags (ESTs), and bacterial artificial chromosome end sequences [17-19]. However, conventional SSR marker development (from enriched libraries) is costly and time consuming [20]. In recent years, with the rapid increase of ESTs in public databases and the advent of bioinformatics tools, SSR marker development has become easier and more cost-effective. Mining SSRs from ESTs is becoming popular for SSR development.

Thanks to global efforts, 11938 SSR markers have been released (CMD website, http://www.cottonmarker.org) up to 2009. Among these SSRs, more than half are EST-SSRs. However, compared to the huge ESTs tank of cotton, only ~25% of the cotton ESTs is applied to SSR development. Cotton ESTs are still a valuable resource for SSR marker development, especially for gene-derived SSRs. It is worth mentioning that the genetic map constructed by Guo et al. contains 71.96% functional marker loci, of which 87.11% are EST-SSR loci [7]. High-density genetic maps of EST-SSR markers are an essential tool for enhanced genome analysis. They represent the transcript part of the genome and can be links between genetic and physical maps [21]. Moreover, as EST-SSRs target coding regions of the genome, they may be useful in association with genes of known function to facilitate the dissection of complex traits [22].

With an endless effort to construct a high-density genetic map of cotton in our laboratory, we have tried RAPDs, SRAPs, and SSRs when no sufficient easy-to-use markers such as SSRs in cotton were available [5,23]. In the last 5 years, the cotton EST project and genome sequencing project generated a great number of sequences that could promote the marker development, and thousands of EST-SSRs and BAC-end SSRs have been developed. Benefited from these projects, we constructed an SSR-based genetic map using SSRs available in 2008 [8]. Thereafter, 700 new Gh-prefixed SSRs [24], new NAU-prefixed SSRs [7,25], and more recently 2937 genomic SSRs (gSSRs) (Monsanto Company) [26] have been released. In this study, we enriched our SSR-based map by these new SSRs; we also isolated and mapped SSRs from ESTs with BLAST hits to known genes, from newly released ESTs of *G. hirsutum *by Yuxian Zhu (GenBank, December 31, 2007), and from newly developed ESTs of *G. barbadense *in our laboratory. Additionally, our recently published markers were also included in this map [27-29], and a final map with 2316 loci map was constructed. This sequence-based, high-density map allowed us to detect segregation distortion regions within the whole genome, to identify gene distribution on chromosomes and homologs between chromosomes.

## Results

### Marker development

A total of 1831 new EST-SSRs were developed from the assembled cotton ESTs in the TIGR database http://www.tigr.org based on the criteria of marker development (see Materials and Methods): 346 from *G. arboretum *(HAU231-HAU576), 293 from *G. raimondii *(HAU577-HAU869), and 1192 from *G. hirsutum *(HAU870-HAU2061).

The 131182 ESTs released by Yuxian Zhu were clustered and assembled into 46296 unique sequences, consisting of 10691 contigs and 35605 singletons. A total of 1047 unique EST-SSRs (HAU2062-HAU3108) were developed from these sequences.

The 10979 ESTs from developing fiber of *G. barbadense *acc. 3-79 generated in our laboratory were clustered and assembled into 5852 unique sequences, consisting of 1492 contigs and 4360 singletons. A total of 299 novel EST-SSRs (HAU3109-HAU3407) were developed from these sequences.

All the marker primers, sequence ID, sequences, motifs, estimated product size, and BLASTX results are listed in Additional file 1.

### Maker polymorphism

Of the 700 Gh-prefixed gSSRs derived from *G. hirsutum*, 134 SSRs (19.1%) showed polymorphism and generated 172 polymorphic loci with an average of 1.28 loci/SSR. Among the 1554 and 754 NAU-prefixed EST-SSRs derived from *G. raimondii *and *G. hirsutum*, respectively, and 578 gSSRs from BAC sequences of *G. hirsutum*, 439 (24.7%), 109 (14.5%), and 68 (11.8%) SSRs were polymorphic, and they generated 537, 131, and 71 loci, respectively, with an average of 1.22 loci/SSR, 1.20 loci/SSR, and 1.0 loci/SSR, respectively.

For these EST-SSRs from the assembled ESTs in TIGR, 28 (8.1%), 33 (13.8%), and 233 (19.5%) SSRs were polymorphic for EST-SSRs from *G. arboretum*, *G. raimondii*, and *G. hirsutum*, respectively, and they generated 29, 36, and 236 loci, respectively. One hundred and sixty-eight SSRs (16.0%) of the 1047 EST-SSRs (HAU2062-HAU3108) were polymorphic and 199 loci were produced; 42 SSRs (14.0%) were polymorphic from the 299 EST-SSRs (HAU3109-HAU3407) and 47 loci were produced.

### Map construction and overview

A total of 1458 loci obtained in this study, adding to the 1026 loci published by Zhang et al. [8] and other published loci [27-29], a total of 2521 loci were applied to map construction. After calculation, 2316 loci including 2311 SSR loci and 5 gene-derived loci were mapped on 26 cotton chromosomes; the total length of this map was 4418.9 cM with an average of 1.91 cM between adjacent markers (see Additional file 2 Figure 1, 2, 3, 4, 5, 6, 7).

**Figure 1:**
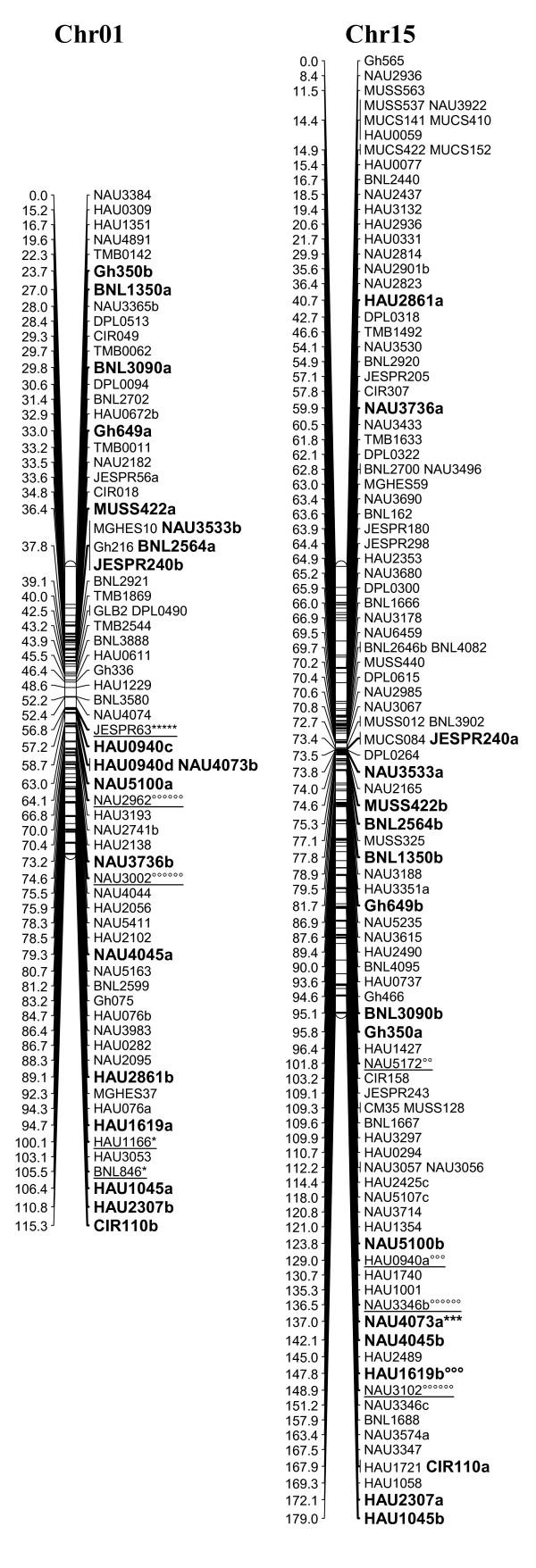
**The genetic map of Chr01/Chr15 homoeologous chromosome**. The interspecific genetic map was constructed using the BC_1 _population [(Emian22 × 3-79) × Emian22]. Duplicated loci are in bold. Map distances are given in centimorgans (cM). Markers showing segregation distortion are underlined and indicated by asterisks (*P < 0.05; ** *P *< 0.01; *** *P *< 0.005; **** *P *< 0.001; ***** *P *< 0.0005; ****** *P *< 0.0001 for markers skewed toward the 'Emian22' allele or degree symbols (°*P *< 0.05; °°*P *< 0.01; °°°*P *< 0.005; °°°°*P *< 0.001; °°°°°*P *< 0.0005; °°°°°°*P *< 0.0001 for markers skewed toward the heterozygous allele). Segregation distortion regions (SDRs) are named as 'Chromosome + No. SDR', for example, SDR2.1 refers to the first SDR on Chr02.

**Figure 2:**
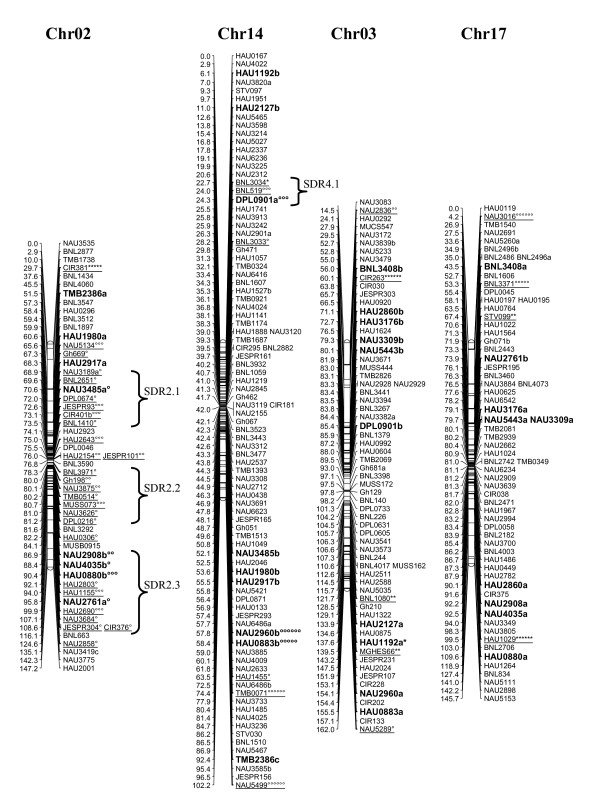
**The genetic maps of Chr02/Chr14 and Chr03/Chr17 homoeologous chromosome**. All legends are same as described for Figure 1.

**Figure 3:**
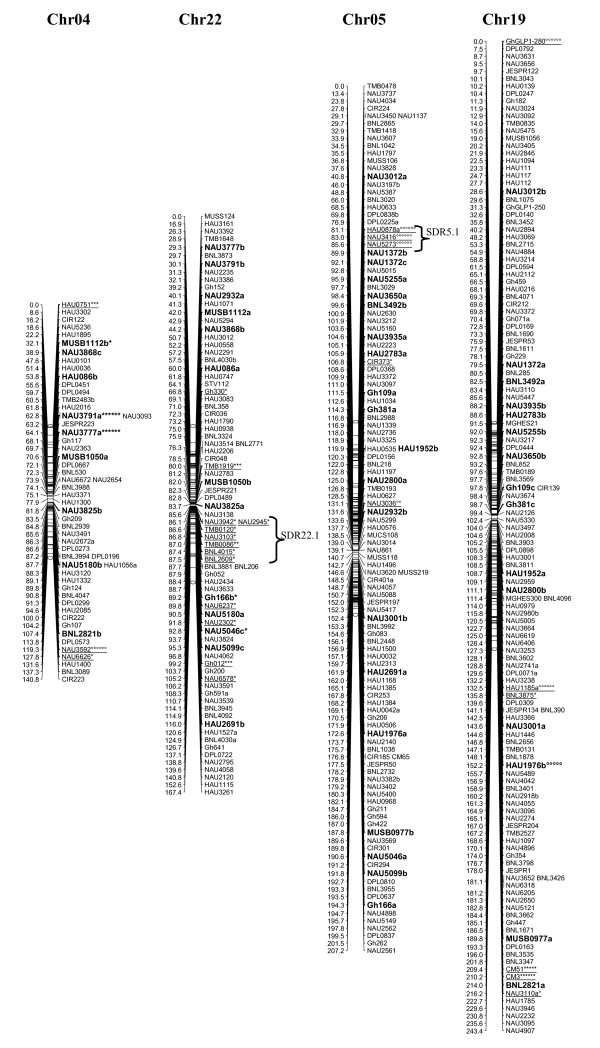
**The genetic maps of Chr04/Chr22 and Chr05/Chr19 homoeologous chromosome**. All legends are same as described for Figure 1.

**Figure 4:**
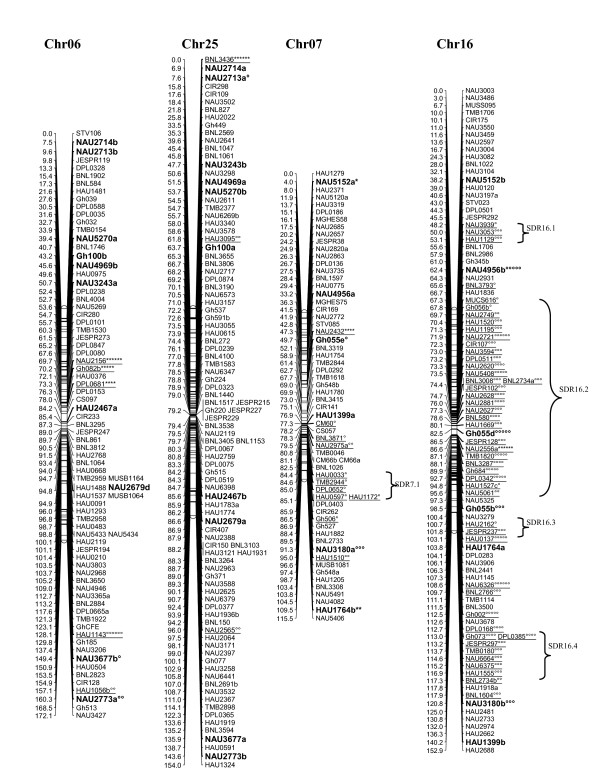
**The genetic maps of Chr06/Chr25 and Chr07/Chr16 homoeologous chromosome**. All legends are same as described for Figure 1.

**Figure 5:**
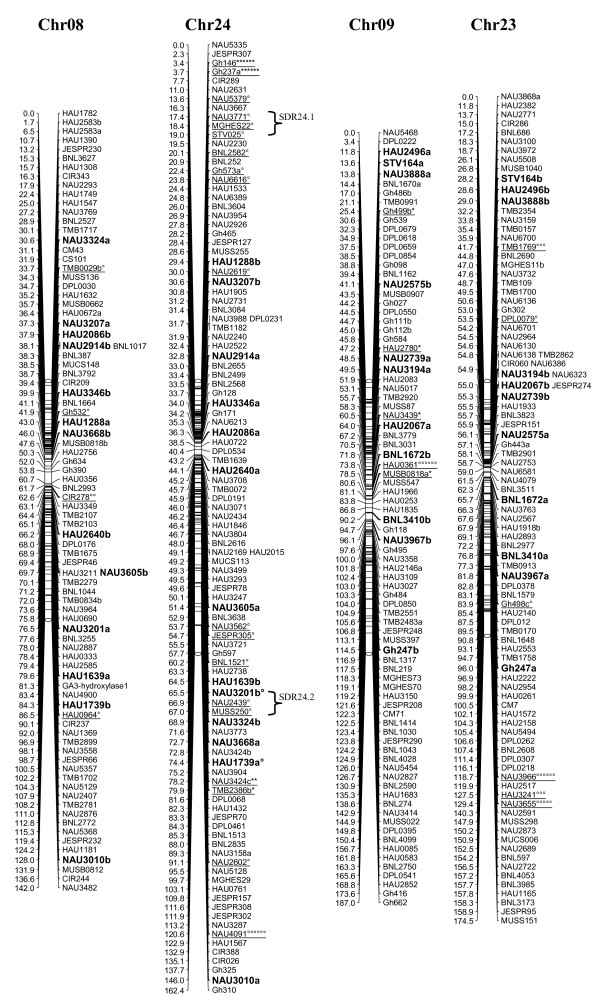
**The genetic maps of Chr08/Chr24 and Chr09/Chr23 homoeologous chromosome**. All legends are same as described for Figure 1.

**Figure 6:**
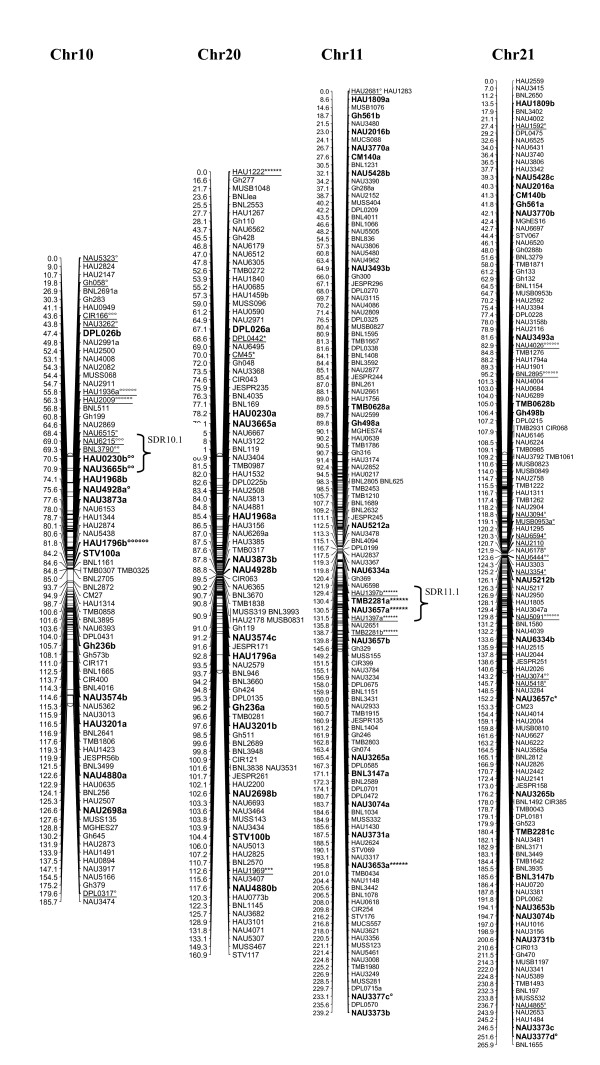
**The genetic maps of Chr10/Chr20 and Chr11/Chr21 homoeologous chromosome**. All legends are same as described for Figure 1.

**Figure 7:**
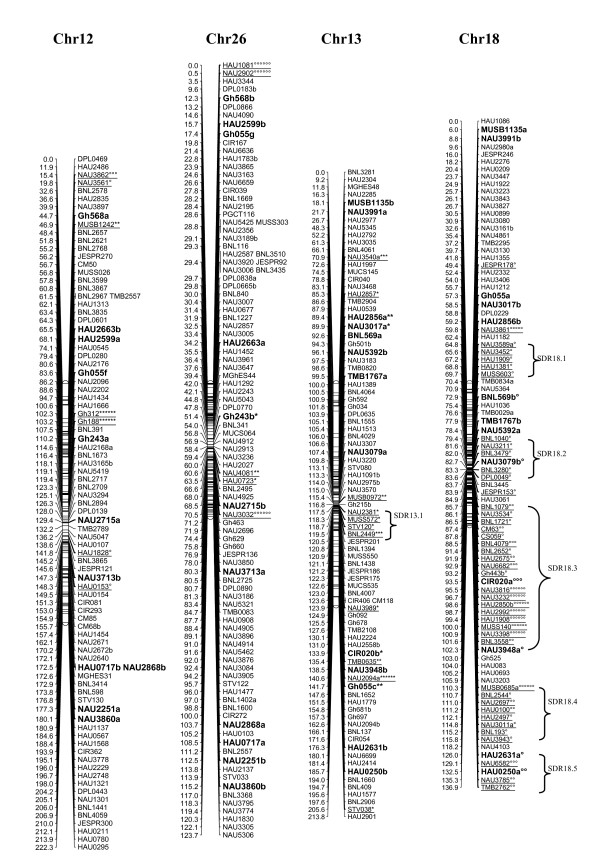
**The genetic maps of Chr12/Chr26 and Chr13/Chr18 homoeologous chromosome**. All legends are same as described for Figure 1.

The chromosome with most loci was Chr19 (134 loci); Chr02 and Chr04 (53 loci) were the chromosomes with the fewest loci. Eighty-nine loci were on each chromosome on average, with 1043 and 1273 loci on At and Dt subgenomes, respectively. More loci were distributed on the Dt subgenome mainly because the NAU-prefixed EST-SSRs were from *G. raimondii *(D_5_), the progenitor of the Dt subgenome of tetraploid cotton [7].

The longest chromosome was Chr21 (265.9 cM) and the shortest was Chr14 (102.2 cM); the average chromosome length was 169.96 cM. The total lengths of the At and Dt subgenomes were 2250.1 cM and 2168.8 cM, respectively, which was the result of more loci on the Dt subgenome to increase recombinants.

The biggest average distance between markers was on Chr02 (2.78 cM) and the least was on Chr14 (1.12 cM). The average distances for At and Dt subgenomes were 2.16 and 1.70 cM, respectively, which also benefited from the more loci on the Dt subgenome. The biggest gap between markers was 23.2 cM on Chr03; there were a total of 35 gaps >10 cM with 15 on At and 20 on Dt subgenome, respectively.

SSRs were not evenly distributed on the cotton chromosomes with more gSSRs and EST-SSRs on the Dt subgenome. More gSSRs were on Chr11, Chr19, and Chr21, and more EST-SSRs on Chr05, Chr11, Chr15, Chr19, Chr21, Chr24, and Chr26. gSSRs and EST-SSRs were also differently distributed on each chromosome; they were similar on Chr02, Chr04, Chr11, Chr19, and Chr20 (difference < 5%), but dramatically different on Chr05, Chr15, Chr18, and Chr26 (difference > 50%).

For different genome-derived SSRs, the A genome-derived SSRs mostly targeted Chr05 and Chr15, and of course preferentially targeted the At subgenome; D genome-derived SSRs mostly targeted Chr05, Chr11, Chr19, Chr21, and Chr26, and also preferentially targeted the Dt subgenome; the AD genome-derived SSRs mostly targeted Chr11, Chr19, and Chr21, but preferentially targeted the Dt subgenome.

### Segregation distortion

Among the 2521 polymorphic loci, 423 loci (16.8%) including one gene-specific locus showed segregation distortion (*P *< 0.05) with 139 loci segregating toward the 'Emian22' allele and 284 loci toward the heterozygous allele. For SSR loci, 15.0% of gSSRs and 18.2% EST-SSRs were distorted, respectively.

A total of 323 distorted loci, accounting for 12.8% of the mapped loci, were mapped on cotton chromosomes with 74.9% segregating toward the heterozygous allele (Figure 1, 2, 3, 4, 5, 6, 7). These segregation distorted loci were unevenly distributed on the 26 cotton chromosomes with 3-51 loci on each chromosome (see Additional file 2). More distorted loci were located on the Dt subgenome than on the At subgenome (195 versus 128). The most distorted loci were on Chr02, Chr16, and Chr18 (> 50% of loci were distorted), and the least on Chr05, Chr08, Chr20, and Chr25 (< 5% of loci were distorted). A total of 21 segregation distortion regions (SDRs) were found on the 26 cotton chromosomes with 8 SDRs on the At subgenome and 13 on the Dt subgenome. More SDRs were found on Chr02, Chr16, and Chr18, the chromosomes with the most distorted loci. The distorted loci showed a phenomenon in which loci skewing toward the same allele appeared on the same chromosomes or within the same SDRs (e.g., Chr02, Chr13, Chr16, and Chr18; Figure 2, 4, 7). Interestingly, adjacent markers in some SDRs showed the same degree of segregation (SDR5.1, SDR7.1, SDR18.1, SDR18.2, SDR24.1, and SDR24.2).

### Annotation and functional characteristics of sequences containing SSRs

In addition to the 1261 EST-SSRs and 5 gene-derived markers, 367 gSSR loci had homologous sequences to cotton ESTs by BLASTN with E ≤ 1 e^-15 ^(see Additional file 3), which indicated that they were transcribed sequences. Thus, a total of 1633 loci of this map (70.6%) were functional markers. The BLASTX results of these transcribed loci showed that 809 loci sequences had no hits to protein; 976, 302, and 54 loci sequences (total 57.5%) had hits to known gene products, hypothetical proteins, and unknown genes, respectively (see Additional file 4).

**Figure 8:**
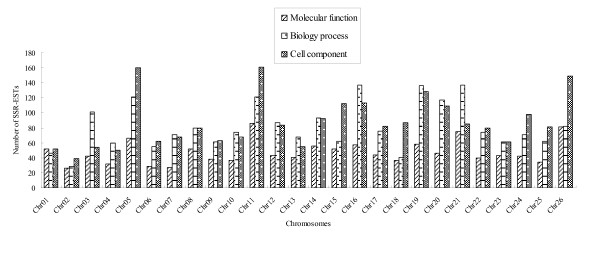
**Chromosome distribution of GO catalogs**.

These sequences containing SSRs totally targeted 38 items of molecular function with 1236 sequences involved in, 85 items of biology process with 2123 sequences involved in and 23 items of cell component with 2273 sequences involved in. However, sequences on different chromosomes targeted different Gene Ontology (GO) catalogs: Chr05, Chr11, Chr21, and Chr26 targeted more molecular functions; Chr03, Chr05, Chr11, Chr16, Chr19, and Chr21 targeted more biology processes; and Chr05, Chr11, and Chr26 targeted more cell components. Most chromosomes targeted more molecular functions and biology processes than cell components, and some chromosomes targeted more special catalogs: Chr03 and Chr21 predominately targeted biology processes and Chr05, Chr11, and Chr26 predominately targeted cell components (Figure 8).

A correlation analysis between gSSRs, EST-SSRs, total loci and GO catalogs showed that GO catalogs were highly correlated to EST-SSR and total loci; gSSR was highly correlated only to the 'biology process'. The results agreed with the concept that functional SSR sequences were mainly derived from EST-SSRs (Table 1).

**Table 1:** Correlation between GO catalogs and gSSRs, EST-SSRs and total loci

GO catalog	gSSR	EST-SSR	Total loci
Molecular function	0.48*	0.77**	0.73**
Biology process	0.60**	0.56**	0.66**
Cell component	0.46*	0.81**	0.74**

*, **: significant at the 0.05 and 0.01 probability levels, respectively.

On level 2 of the GO classification, 'binding' (48.95%) and 'catalytic activity' (30.87) dominated the molecular function; 'metabolic process' (27.82%) and 'cellular process' (27.69%) dominated 50% of the biology process; and 'cell part' (32.86%), 'cell' (32.86%), and 'organelle' (25.27%) predominated the cellular component (see Additional file 5).

On level 3 of the GO classification, 'nucleic acid binding', 'ion binding', 'transferase activity', 'protein binding', 'nucleotide binding', and 'hydrolase activity' predominated in nearly 70% of the molecular function; 'cellular metabolic process', 'primary metabolic process', 'macromolecule metabolic process', and 'biosynthetic process' took more than 50% of the biology process; and 'intracellular', 'intracellular part', 'intracellular organelle', and 'membrane-bounded organelle' were major items of cell component (see Additional file 6).

When taking individual chromosomes into account, we found that some GO items were found only in some chromosomes, for example, Chr08 occupied many special GO items of biology process. Also, some chromosomes dominated some GO items, for example, Chr05 and Chr26 dominated the four major items of cell component (see Additional file 7).

## Discussion

High-density genetic maps are becoming increasingly important in theoretical and applied genetic research [30]. The construction of genetic maps in cotton with molecular markers has been hampered by the limited polymorphism which is 18.2%-47.9% between *G. hirsutum *and *G. barbadense *[5,7,31-33] and even less (4.13%-7.9%) among *G. hirsutum *germplasms [12,13,34,35]. Due to the low polymorphism and the large genome size in cotton, the only way to construct a high-density genetic linkage map is to apply more markers. In light of this, we developed new SSRs from ESTs annotated to proteins and newly released ESTs including novel ESTs from *G. barbadense *acc. 3-79 developed in our laboratory. As a result, 3177 new EST-SSRs were developed.

These EST-SSRs showed a lower polymorphism ratio (8.1%-19.5%) when compared with other researches [7,31-33]. Among the NAU-prefixed EST-SSRs and gSSRs, only half of the polymorphism was detected compared to the results of Guo et al. [7,25]. Although our population and the population described by Guo et al. [7] are both interspecific populations, a different polymorphism was found between the two populations, which might result from different materials or different genotyping methods (denatured polyacrylamide gel vs. non-denatured polyacrylamide gel).

The 6185 SSR primers developed in this study generated 1458 loci, and with our previous data [8], a total of 2521 loci were applied to map construction. The resulting map included 2316 loci on the 26 cotton chromosomes, 4418.9 cM long with an average distance of 1.91 cM between adjacent markers. This map is one of the three maps composed of more than 2000 markers. Compared to the map published by Rong et al. (2584 loci, 4447.9 cM in length with an average distance of 1.72 cM) [3] and the map published by Guo et al. (2247 loci, 3440.4 cM in length with an average distance of 1.58 cM) [25], the total map length and the average marker distance of our map were more similar to the map of Rong et al. [3], but longer than that of Guo et al. [25]. The marker distribution on chromosomes was similar to the map of Guo et al. [7] with the most loci on Chr19 and the fewest on Chr02 and Chr04. More loci were found on the Dt subgenome than on the At subgenome in our map and the map of Guo et al. [7], but more loci were present on the At subgenome in the map of Rong et al. [3]. The At subgenome was longer than the Dt subgenome in our map and the map of Rong et al. [3], but shorter in the map of Guo et al. [7]. The average marker distance of the At subgenome is longer than that of the Dt subgenome in all the three maps. The number of gaps (> 10 cM) in our map was similar to the map of Guo et al. [7], but fewer than shown in the map of Rong et al. [3]; there were more gaps on the Dt subgenome than on the At subgenome in our map, but fewer in the other maps.

By comparing the distribution of SSRs, we found that both gSSRs and EST-SSRs were prone to be mapped on the Dt subgenome, with more gSSRs and EST-SSRs on Chr11, Chr19, and Chr21. The distributions of gSSRs and EST-SSRs on each chromosome were also different with four chromosomes having 50% more EST-SSRs. The uneven distribution of SSRs on subgenomes and chromosomes could help us to determine the distribution of SSR motifs; and the function of SSR sequences could be conducive to further target genes in the cotton genome. The distribution of different genome-derived SSRs can provide us with some evidence for the evolution of tetraploid cotton; for example, Chr05, Chr11, and Chr15 may undergo concerted evolution because of the distributions of other genome-derived SSRs on them.

Segregation distortion is widespread in plant populations. In this study, 16.8% of the total loci showed segregation distortion (*P *< 0.05), which is similar to other reports on cotton (12.3%-19.9%) [6,7,23]. Twice as many loci segregated toward the heterozygous allele than the 'Emian22' allele, and EST-SSRs showed more segregation distortion than gSSRs did. For the mapped loci, 12.8% of them were mapped on cotton chromosomes with 74.9% segregating toward the heterozygous allele. The loci segregated toward the heterozygous allele with a high frequency because the heterozygous allele could not be distinguished from the '3-79' allele in the BC_1 _population. Three chromosomes (Chr02, Chr16, and Chr18) showed extreme segregation distortion in that >50% of loci were distorted, among which 99.9% of the distorted loci segregated toward the heterozygous allele (Figures 2, 4, 7). Other maps have also proved that these chromosomes show more segregation distortion [3,6,7]. The extremely distorted chromosomes indicated to us that segregation distortion loci exist on these chromosomes. Faris et al. [36] and Kumar et al. [37] used reciprocal backcross populations to identify segregation distortion loci in *Aegilops tauschii *and tetraploid wheat, respectively, which provided us with an example for identifying segregation distortion loci in cotton. The loci skewing toward the same allele clustered on the same chromosomes or within the same SDRs indicated that genetic hitchhiking effects occur in cotton.

More than 8000 EST-SSRs were used in our mapping population; however, only 1261 EST-SSRs were mapped. Although gSSRs derived from genomic sequences, their mother sequences can also be transcribed or translated. By blasting the gSSR sequences to cotton ESTs, 367 gSSR sequences were matched to cotton ESTs. As a result, 1633 loci of this map (70.6%) including five gene-derived markers were functional markers, which was fewer than those reported by Guo et al. [7]. By blasting to the protein database, 1332 loci were derived from translated sequences. Functional annotation of these loci sequences revealed that some chromosomes preferentially targeted certain GO catalogs, specifically, Chr03 and Chr21 mostly targeted the biology process; some GO items were found only on some chromosomes, that is, many special GO items of the biology process were detected only on Chr08. These results indicated that some chromosomes in cotton perform special functions. What's more, because these ESTs were mainly developed from developing fibers, this map can also be used to identify fiber-related genes and to detect expressed QTLs (eQTLs) for fiber quality.

## Conclusions

A total of 3177 new EST-SSRs were developed to enrich our interspecific BC_1 _genetic linkage map. The final map included 2316 loci on the 26 cotton chromosomes, 4418.9 cM in total length and 1.91 cM in average distance between adjacent markers. Segregation distorted chromosomes were identified, which is a guide to identify segregation distortion loci in cotton and to understand the mechanism of segregation distortion in interspecific cross between *G. hirsutum *and *G. barbadense *in cotton. SSR sequences were functionally annotated, which is helpful to identify functions of cotton chromosomes and to detect eQTLs for fiber quality. This map can be compared and integrated with other cotton maps to construct a consensus map in cotton.

## Methods

### Plant materials

The mapping population used in this study is the BC_1 _population [(Emian22 × 3-79) × Emian22] including 141 individuals which had been used to construct a 917-locus map [8]. 'Emian22' is an upland cotton cultivar with high yield and moderate fiber quality but not resistant to verticillium wilt in Hubei Province. It was once widely cultivated and is still used as a parent for hybrid production. Sea-island cotton accession '3-79' is the genetic and cytogenetic standard line for *G. barbadense *with super fiber quality and high resistance to verticillium wilt. The cross between these two materials is performed to improve the performance of 'Emian22' by backcrossing and molecular-assisted selection.

### EST-SSR marker development

To effectively identify SSRs in ESTs, a Windows-based tool named Serafer was developed in our laboratory, available at ftp://ensembl.genomics.org.cn/other/Serafer_1.9.5.zip. Serafer combines CAP3 [38], Sputnik (Abajian, Washington University; http://espressosoftware.com/sputnik), and Primer3.0 [39] into a package; it can assemble the sequences, search SSRs, and design primers at the same time, so it is a powerful pipeline for SSR identification. Serafer takes a FASTA formatted sequence file as an input and can produce an Excel file with number of SSRs, sequence ID, SSR type, SSR motif, SSR position, repeat length, repeat number, repeat score, and the length of the sequence.

The assembled cotton EST sequences (*G. arboretum *release 2, *G. raimondii *release 2, and *G. hirsutum *release 3) were downloaded in FASTA format from the Institute for Genome Research (TIGR) database http://www.tigr.org. The default criteria for SSR detection were a minimum of seven repeats for dinucleotide motifs, five repeats for trinucleotide motifs, and four repeats for tetra-, penta-, and hexanucleotide motifs. The contig or singleton sequences were used to design primers flanking the putative SSRs. The input criteria for primer design were primer length 18-24 bp with 20 bp as the optimum, GC content 35%-60% with 50% as the optimum, optimum annealing temperature 57°C, and PCR product size 100-300 bp. After the SSR identification and primer design for the three sets of data were completed, three steps were conducted to generate the final unique SSR markers. First, EST-SSRs (ESTs containing SSR) with no BLAST hits to known genes were excluded; second, unique SSRs for each dataset were generated by comparing them to all public cotton microsatellites deposited in CMD http://www.cottonmarker.org according to sequence ID, target SSR, and primer sequences; and third, internal companions among the three datasets were conducted to reduce redundancies. This work was finished as of January 2008.

The ESTs from fast-elongating fiber of *G. hirsutum *cv. Xu-142 released by Yuxian Zhu were the second sequence source for EST-SSRs development, which was not included in the assembled ESTs of *G. hirsutum *release 3 in TIGR. These sequences were analyzed in the Serafer pipeline from sequence assembly to primer design. More strict criteria were set for SSR detection with a minimum length of 18 bp for di- to hexanucleotide motifs, and the parameters for primer design were the same as before except that the PCR product size was 100-400 bp. These SSR markers were also compared to all existing markers by primer sequences to ensure that they were not redundant. This process was finished as of July 2008.

The third sequence data for the development of EST-SSRs were the new ESTs from developing fiber of *G. barbadense *acc. 3-79 generated in our laboratory (unpublished data). Aiming at understanding the mechanism of fiber development in *G. barbadense*, we have constructed a normalized fiber cDNA library (from -2 to 25 dpa) of *G. barbadense *acc. 3-79 [40]. A preliminary sequencing of the cDNA library produced 887 high-quality ESTs that were used to construct the transcript map of developing fiber [40] and also to isolate EST-SSRs [41]. After that, an additional 10090 high quality ESTs were obtained. These new sequences combined with the previous sequences were explored in the Serafer pipeline to develop SSR markers. The SSR markers that were the same as all the previously developed SSRs were excluded from the results, and this was finished as of August 2008.

All the SSR primers were named with a prefix HAU and synthesized by Sunbiotech Co. Ltd. (Beijing).

### Marker analysis

PCR, electrophoresis and silver staining were performed as previously described [23].

### Map construction

The mapping data for each parent were scored as the BC_1 _data according to the definition of JoinMap 3.0 [42], and the linkage map was constructed using a logarithm of odds (LOD) threshold of 5.0 and a maximum recombination fraction of 0.4. Map distances in centi-Morgans (cM) were calculated using the Kosambi mapping function [43]. The resulting linkage map was drawn using MapChart 2.2 software [44]. Linkage groups were assigned to corresponding chromosomes by mapped SSRs http://www.cottonmarker.org. Homoeologous chromosomes were identified by duplicated loci as described in previous reports [2,3,7].

### Segregation distortion

For each segregating marker, a χ^2 ^analysis was performed to test for deviation from the 1:1 expected segregation ratio. A region with at least three adjacent loci showing significant segregation distortion (*P *< 0.05) was defined as the segregation distorted region (SDR) [45].

### Annotation and functional classification of sequences containing SSRs

Sequences corresponding to gSSRs were indentified to be transcribed sequences by BLASTN to cotton ESTs with E ≤ 1 e^-15 ^and were annotated using BLASTX (NCBI, Bethesda, MD, USA). The BLASTX results were classified into three groups: known gene products, hypothetical proteins, and unknown genes.

Functional annotation of ESTs was based on GO annotation [46], and performed with BLAST2GO [47,48]. When running BLAST2GO, BLASTX DB was set to NCBI non-redundant DB and expectation value threshold was set at 1.0E-3, whereas high-scoring segment pairs (HSPs) length cutoff was set at 15. The top 20 BLAST hits were retained, then go-mapping was run, and an annotation step with default parameters was performed. Furthermore, InterPro Scan was performed and InteProScan GOs was merged to annotation. Finally, the 'goslim_plant.obo' ontology subset was used to achieve specific GO terms.

## Authors' contributions

YY carried out most of the genotyping of the BC_1 _population. DJY carried out the bioinformatics analysis of the functional annotation of these SSR sequences. SGL developed the SSR analysis pipeline. XML participated in part of the genotyping of the BC_1 _population. XQW also participated in part of the genotyping of the BC_1 _population. ZXL designed the study, conducted the data analysis and drafted the manuscript. XLZ participated in the design of the study and proofread the manuscript. All authors contributed to the interpretation of the results, read and approved the final manuscript.

## Supplementary Material

Additional file 1 **EST-SSRs developed from cotton ESTs (The details of marker name, sequence ID, sequence, motif, primer sequences, product size, Tm and putative function are listed**). Click here for file

Additional file 2 **Chromosome assignment, marker distribution, length of chromosomes, marker density, gaps and segregation distortion in genetic linkage map constructed with the [(Emian22 × 3-79) × Emian22] BC**_1 _**population**. Click here for file

Additional file 3 **gSSRs derived from transcribed sequences**. Click here for file

Additional file 4 **Description: BLASTX results of SSR sequences**. Click here for file

Additional file 5 **GO classification of mapped loci sequences (level 2): (a) molecular function; (b) cell component; and (c) biology process**. Click here for file

Additional file 6 **GO items of mapped loci sequences (level 3): (a) molecular function; (b) cell component; and (c) biology process**. Click here for file

Additional file 7 **Details of GO results**. Click here for file
