# Supplementary material for: Genome structure of cotton revealed by a genome-wide SSR genetic map constructed from a BC1 population between gossypium hirsutum and G. barbadense
Source: BMC Genomics. 2011 Jan 9;12:15. doi: 10.1186/1471-2164-12-15 (PMC3031231; doi:10.1186/1471-2164-12-15)
Supplement: Additional file 2 — Chromosome assignment, marker distribution, length of chromosomes, marker density, gaps and segregation distortion in genetic linkage map constructed with the [(Emian22 × 3-79) × Emian22] BC1 population. [file 1471-2164-12-15-S2.DOC]

**Table S2 Chromosome assignment, marker distribution, length of chromosomes, marker density, gaps and segregation distortion in genetic linkage map constructed with the [(Emian22 × 3-79) × Emian22] BC_1_ population**

| Chromosome | gSSR | EST-SSR | Genome-derived SSRs | | | Total loci | Length (cM) | cM/marker | >10 cM  gap | SD loci | SD% | SDRs |
| --- | --- | --- | --- | --- | --- | --- | --- | --- | --- | --- | --- | --- |
|  |  |  | A | D | AD |  |  |  |  |  |  |  |
| Chr01 | 28 | 41 | 3 | 18 | 48 | 70 | 115.3 | 1.65 | 1 | 5 | 7.1 | 0 |
| Chr02 | 27 | 26 | 3 | 13 | 37 | 53 | 147.2 | 2.78 | 2 | 32 | 60.4 | 3 |
| Chr03 | 27 | 38 | 5 | 19 | 41 | 65 | 162.0 | 2.49 | 2 | 6 | 9.2 | 0 |
| Chr04 | 27 | 26 | 0 | 13 | 40 | 53 | 140.8 | 2.66 | 0 | 6 | 11.3 | 0 |
| Chr05 | 42 | 74 | 11 | 43 | 62 | 116 | 207.2 | 1.79 | 3 | 5 | 4.3 | 1 |
| Chr06 | 42 | 35 | 4 | 18 | 55 | 78 | 172.1 | 2.21 | 1 | 7 | 9.0 | 0 |
| Chr07 | 30 | 32 | 0 | 16 | 46 | 62 | 115.5 | 1.86 | 0 | 14 | 22.2 | 1 |
| Chr08 | 39 | 46 | 5 | 19 | 61 | 86 | 142.0 | 1.65 | 0 | 4 | 4.7 | 0 |
| Chr09 | 49 | 35 | 6 | 13 | 65 | 84 | 187.0 | 2.23 | 1 | 5 | 6.0 | 0 |
| Chr10 | 35 | 42 | 3 | 19 | 55 | 77 | 185.7 | 2.41 | 2 | 14 | 18.2 | 1 |
| Chr11 | 63 | 63 | 9 | 37 | 80 | 126 | 239.2 | 1.90 | 0 | 8 | 6.3 | 1 |
| Chr12 | 40 | 47 | 6 | 16 | 65 | 87 | 222.3 | 2.56 | 2 | 7 | 8.0 | 0 |
| Chr13 | 41 | 45 | 6 | 13 | 67 | 86 | 213.8 | 2.49 | 1 | 15 | 17.4 | 1 |
| At | 490 | 550 | 61 | 257 | 722 | 1043 | 2250.1 | 2.16 | 15 | 128 | 12.3 | 8 |
| Chr14 | 36 | 55 | 1 | 29 | 61 | 91 | 102.2 | 1.12 | 0 | 9 | 9.9 | 1 |
| Chr15 | 35 | 70 | 14 | 32 | 59 | 105 | 179.0 | 1.70 | 0 | 6 | 5.7 | 0 |
| Chr16 | 40 | 51 | 2 | 29 | 60 | 91 | 152.9 | 1.68 | 1 | 51 | 56.0 | 4 |
| Chr17 | 27 | 37 | 1 | 21 | 42 | 64 | 145.7 | 2.28 | 2 | 4 | 6.3 | 0 |
| Chr18 | 32 | 56 | 4 | 30 | 54 | 88 | 136.9 | 1.56 | 0 | 47 | 53.4 | 5 |
| Chr19 | 64 | 68 | 2 | 39 | 91 | 134 | 243.4 | 1.82 | 0 | 7 | 5.2 | 0 |
| Chr20 | 49 | 47 | 5 | 23 | 68 | 96 | 160.9 | 1.68 | 4 | 4 | 4.2 | 0 |
| Chr21 | 64 | 69 | 1 | 41 | 91 | 133 | 265.9 | 2.00 | 2 | 15 | 11.3 | 0 |
| Chr22 | 33 | 45 | 2 | 25 | 51 | 78 | 167.4 | 2.15 | 4 | 15 | 19.2 | 1 |
| Chr23 | 49 | 45 | 4 | 25 | 65 | 94 | 174.5 | 1.86 | 3 | 6 | 6.4 | 0 |
| Chr24 | 44 | 61 | 3 | 34 | 68 | 105 | 162.4 | 1.55 | 2 | 21 | 20.0 | 2 |
| Chr25 | 53 | 42 | 0 | 21 | 74 | 95 | 154.0 | 1.62 | 2 | 4 | 4.2 | 0 |
| Chr26 | 34 | 65 | 2 | 42 | 55 | 99 | 123.7 | 1.25 | 0 | 6 | 6.1 | 0 |
| Dt | 560 | 711 | 41 | 391 | 839 | 1273 | 2168.8 | 1.70 | 20 | 195 | 15.3 | 13 |
| Total | 1050 | 1261 | 102 | 648 | 1561 | 2316 | 4418.9 | 1.91 | 35 | 323 | 13.9 | 21 |
