# Supplementary figures and images for: Genome structure of cotton revealed by a genome-wide SSR genetic map constructed from a BC1 population between gossypium hirsutum and G. barbadense
Source: BMC Genomics. 2011 Jan 9;12:15. doi: 10.1186/1471-2164-12-15 (PMC3031231; doi:10.1186/1471-2164-12-15)

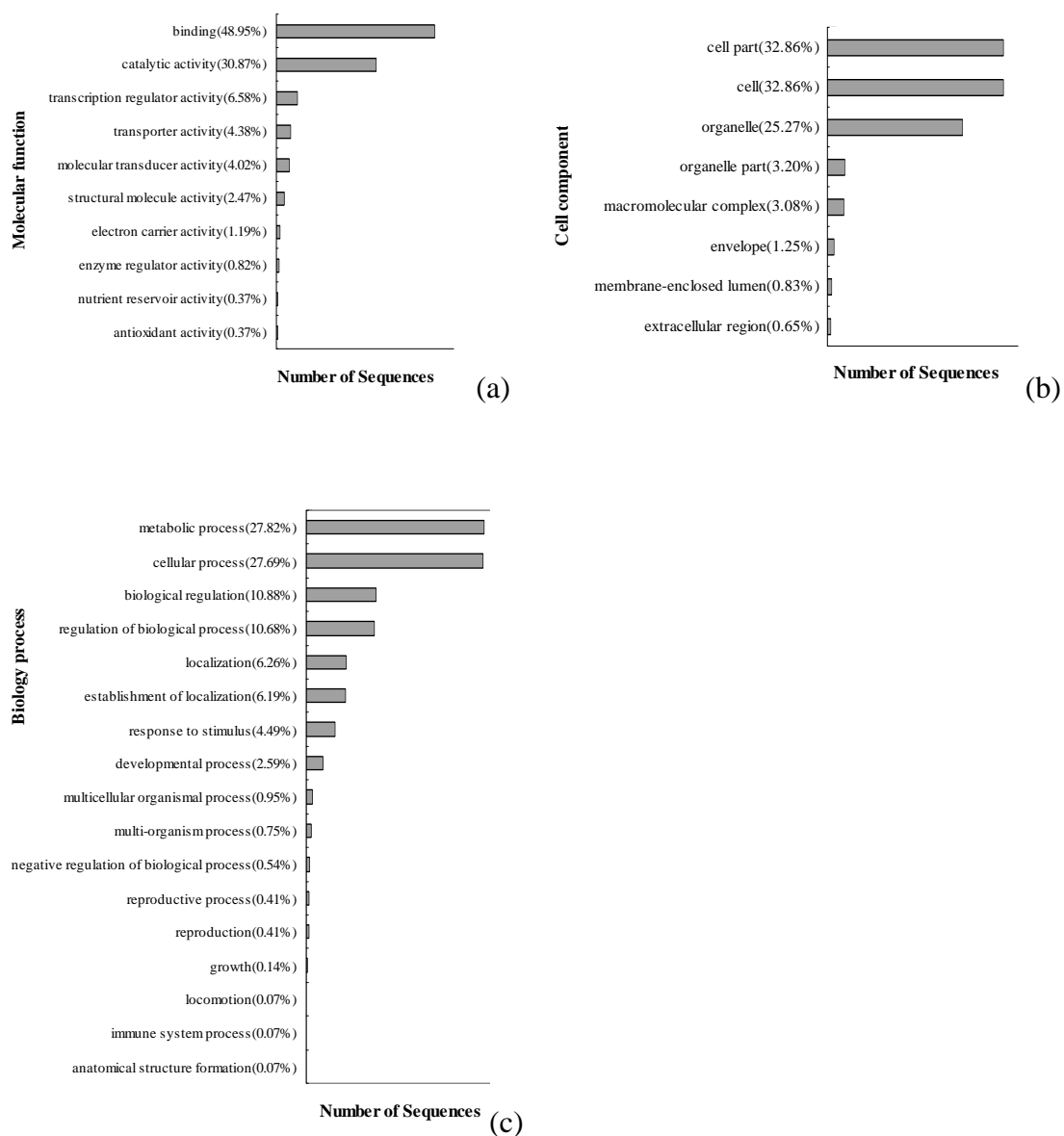

**Figure S1**

Supplement: Additional file 5 — GO classification of mapped loci sequences (level 2): (a) molecular function; (b) cell component; and (c) biology process. [file 1471-2164-12-15-S5.PDF]

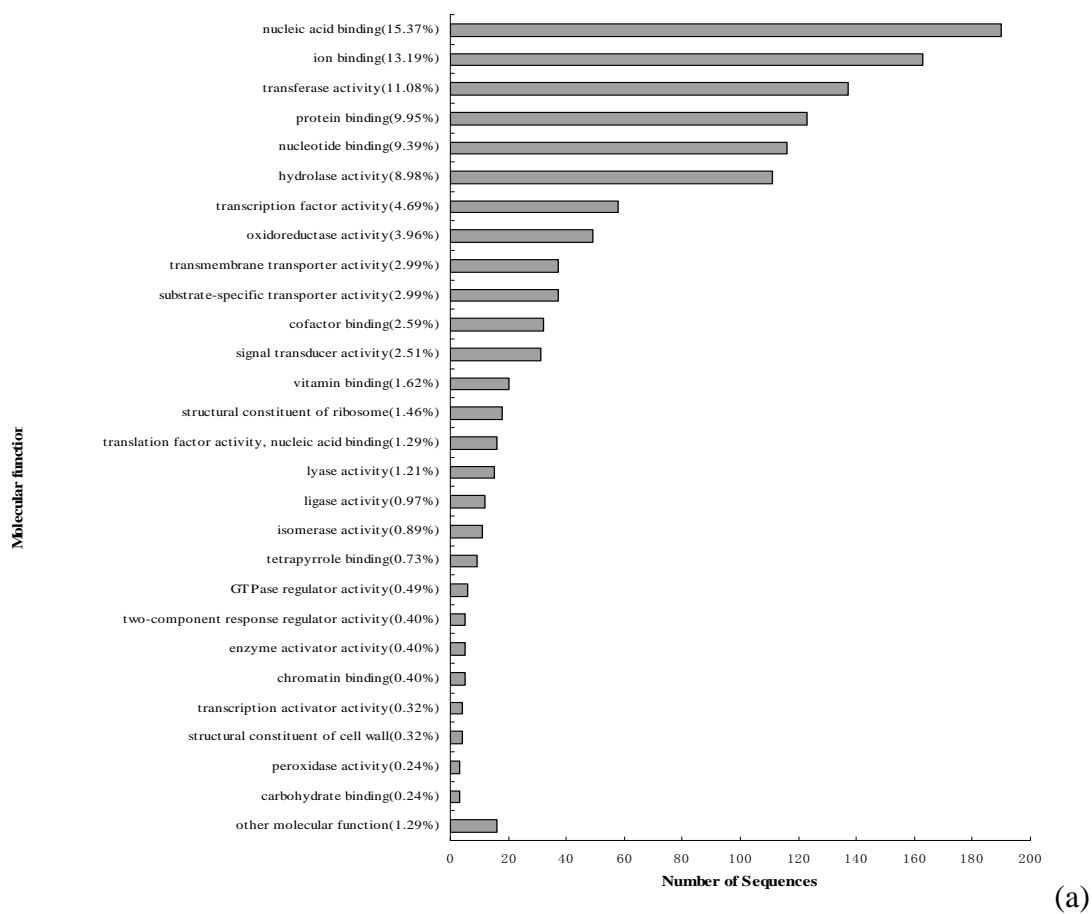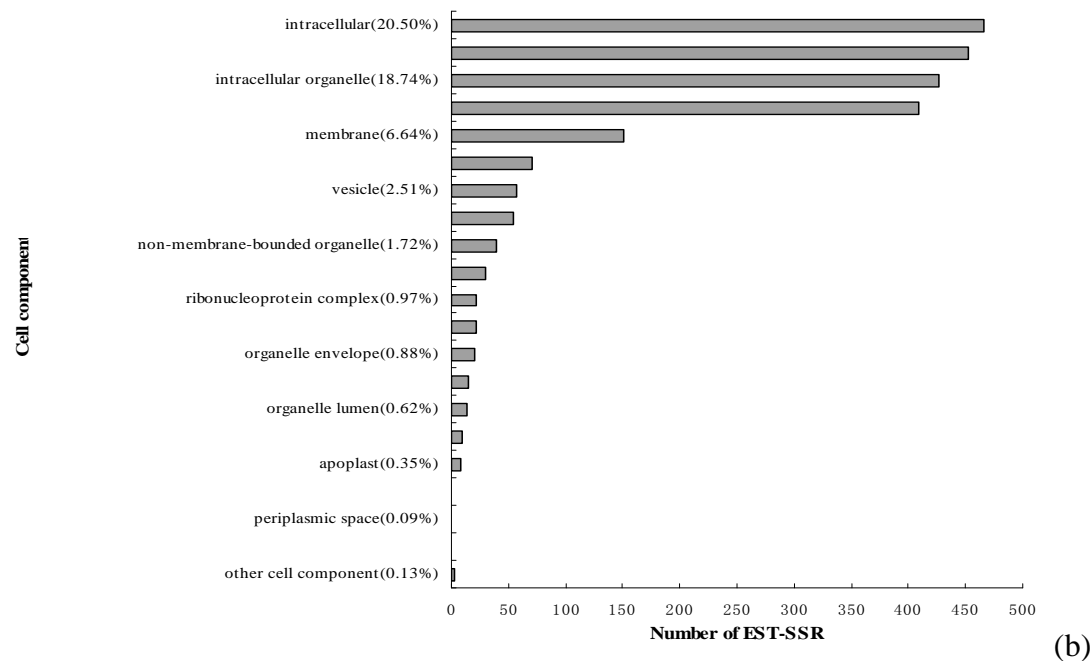

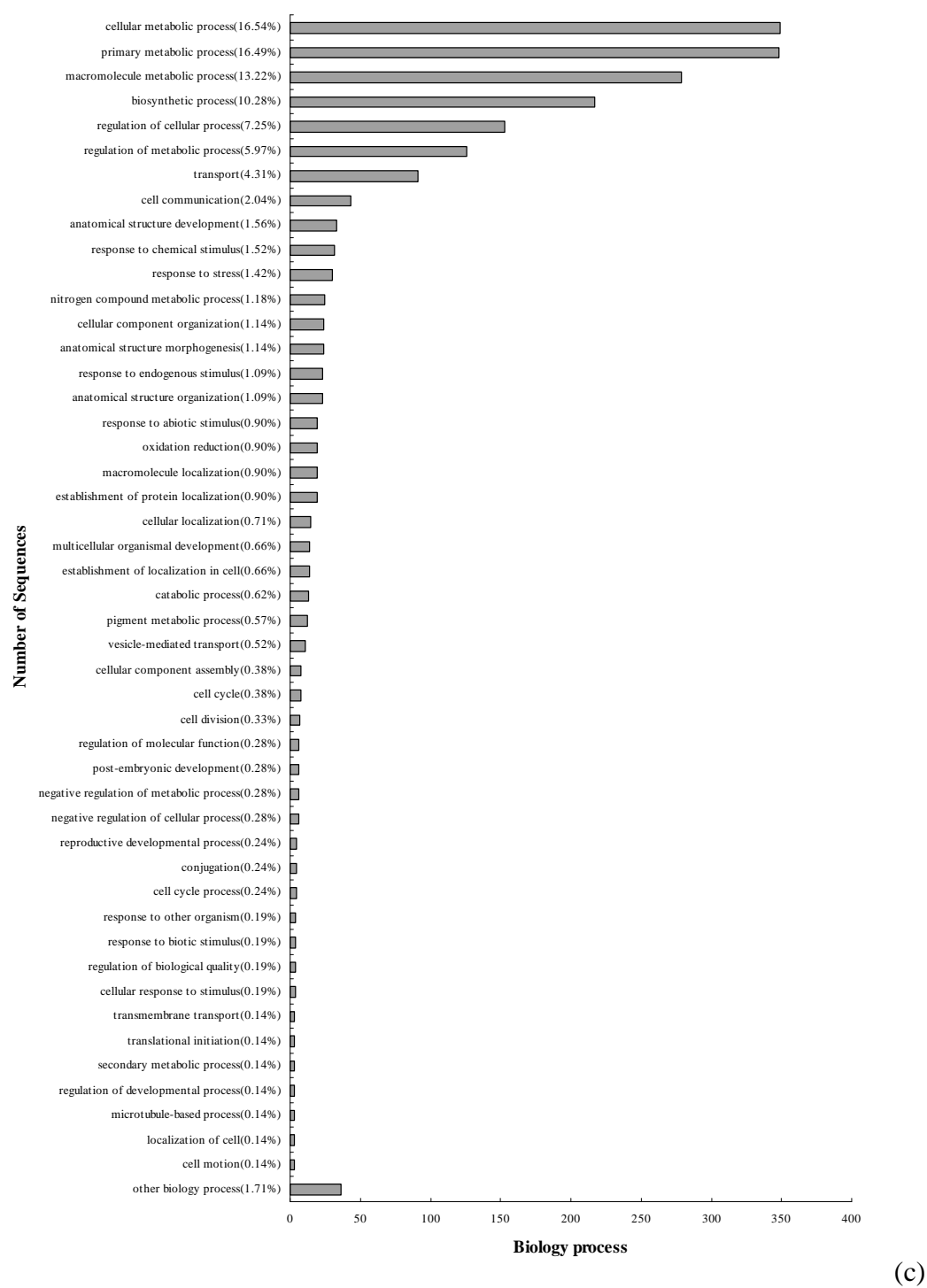

**Figure S2**

Supplement: Additional file 6 — GO items of mapped loci sequences (level 3): (a) molecular function; (b) cell component; and (c) biology process. [file 1471-2164-12-15-S6.PDF]
